# MC1568 improves insulin secretion in islets from type 2 diabetes patients and rescues β-cell dysfunction caused by Hdac7 upregulation

**DOI:** 10.1007/s00592-018-1201-4

**Published:** 2018-08-07

**Authors:** Mahboubeh Daneshpajooh, Lena Eliasson, Karl Bacos, Charlotte Ling

**Affiliations:** 1 Epigenetics and Diabetes Unit, Department of Clinical Sciences, Lund University Diabetes Centre, CRC 91:12, Box 50332, 20213 Malmö, Sweden; 2 Islet Cell Exocytosis Unit, Department of Clinical Sciences, Lund University Diabetes Centre, CRC 91:11, Box 50332, 20213 Malmö, Sweden

**Keywords:** Type 2 diabetes, Insulin secretion, MC1568, Human pancreatic islets, HDAC inhibitor, Epigenetics

## Abstract

**Aims:**

It has in recent years been established that epigenetic changes contribute to β-cell dysfunction and type 2 diabetes (T2D). For example, we have showed that the expression of histone deacetylase 7 (HDAC7) is increased in pancreatic islets of individuals with T2D and that increased levels of Hdac7 in β-cells impairs insulin secretion. The HDAC inhibitor MC1568 rescued this secretory impairment, suggesting that inhibitors specific for HDAC7 may be useful clinically in the treatment of T2D. The aim of the current study was to further explore HDAC7 as a novel therapeutic target in T2D.

**Methods:**

Hdac7 was overexpressed in clonal β-cells followed by the analysis of insulin secretion, mitochondrial function, as well as cell number and apoptosis in the presence or absence of MC1568. Furthermore, the effect of MC1568 on insulin secretion in human pancreatic islets from non-diabetic donors and donors with T2D was also studied.

**Results:**

Overexpression of Hdac7 in clonal β-cells significantly reduced insulin secretion, mitochondrial respiration, and ATP content, while it increased apoptosis. These impairments were all rescued by treatment with MC1568. The inhibitor also increased glucose-stimulated insulin secretion in islets from donors with T2D, while having no effect on islets from non-diabetic donors.

**Conclusions:**

HDAC7 inhibition protects β-cells from mitochondrial dysfunction and apoptosis, and increases glucose-stimulated insulin secretion in islets from human T2D donors. Our study supports specific HDAC7 inhibitors as novel options in the treatment of T2D.

## Introduction

Both genetic and epigenetic variations contribute to impaired pancreatic islet function and type 2 diabetes (T2D) [1, 2]. Studies have also implicated epigenetic regulators such as histone deacetylases (HDACs) in the development and function of β-cells, thus supporting the use of HDAC inhibitors for the treatment of diabetes. For example, Hdac3 is involved in β-cell development [3], while both genetic and pharmacological inhibition of Hdac3 improves adult β-cell survival and function in rodents [4–7]. In addition, knockout of *Hdac5* or *-9* leads to an increased pool of insulin producing β-cells [8] and the HDAC inhibitor valproic acid increases β-cell numbers and function in streptozotocin treated rats [9]. Recently, we reported that *HDAC7* expression is upregulated in pancreatic islets from subjects with T2D and that increased Hdac7 levels impair insulin secretion in both isolated rodent islets and clonal β-cells. Furthermore, pharmacological and genetic inhibition of Hdac7 rescued the defects in insulin secretion [10]. The present study aimed to further explore the promise of HDAC7 as a novel therapeutic target in treatment of T2D via evaluating the effects of the HDAC inhibitor MC1568 in clonal β-cells overexpressing Hdac7 and islets from donors with T2D.

## Methods

### Human islets

Pancreatic islets were obtained from the Nordic Network for Islet Transplantation, Uppsala University, Sweden. Informed consent was obtained from pancreatic donors or their relatives in accordance with the approval by the local ethics committee regarding organ donation for medical research. Islets were hand-picked and randomly selected for treatment with 1 µmol/l MC1568 for 24 h after which the inhibitor was removed. Islet were then preincubated in secretion assay buffer (SAB) [10] containing 2.8 mmol/l glucose for 1 h (8–12 islets/well, 5–10 wells per condition). All islets in each well were then randomly transferred to new plates containing fresh SAB with 2.8 or 16.7 mmol/l glucose, and incubated for 1 h at 37 °C. The buffer was harvested and islets lysed for the extraction of insulin content. Insulin in buffer and lysate was measured with an ELISA (Mercodia, Sweden), and secretion was normalized to total insulin content.

### *Hdac7* overexpression in clonal β-cells

Hdac7 with or without a c-terminal HA-tag was overexpressed in rat INS-1 832/13 β-cells, kindly shared with us by Professor Christopher Newgard at Duke University, before the determination of insulin secretion, ATP content, mitochondrial respiration, cell number, and apoptosis. All experiments were performed as described [10]. Treatment with the MC1568 HDAC inhibitor was started 24 h post-transfection, and the inhibitor was removed before experiments were initiated.

### Statistical analysis

Wilcoxon signed-rank tests were used to analyze the clonal β-cell data. A paired *t* test was used to analyze the insulin secretion data within the groups of non-diabetic donors and donors with T2D, while a Mann–Whitney test was used to analyze the data within each donor separately. Data are reported as mean ± SEM.

## Results

### MC1568 rescues β-cell defects caused by *Hdac7* overexpression

**Fig. 1:**
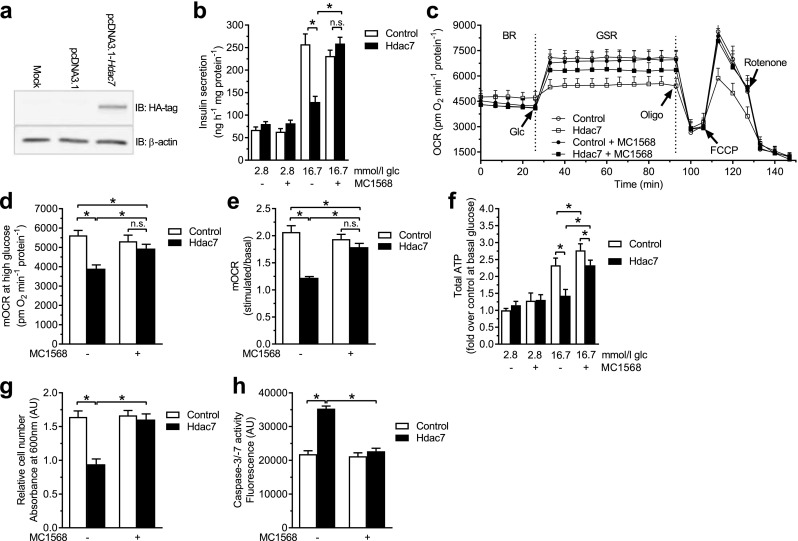
MC1568 rescues β-cell impairments induced by Hdac7 overexpression. **a** Western blot with a primary antibody against the HA-tag showed that Hdac7 was overexpressed after transfection with pcDNA3.1-*Hdac7*-HA. **b** Hdac7 overexpression for 48 h resulted in impaired glucose-stimulated insulin secretion compared with control transfected cells (pcDNA3.1 plasmid). Treatment with 1 µmol/l MC1568 completely rescued this phenotype (*n* = 6). **c** Oxygen consumption rate (OCR) measured in real time in Hdac7 and control transfected clonal β-cells with and without MC1568 treatment. The OCR was measured in the presence of 2.8 mmol/l glucose (basal respiration, BR) and then after the sequential addition of 16.7 mmol/l glucose (glucose-stimulated respiration, GSR), 4 µg/ml of oligomycin (oligo), 4 µmol/l FCCP (carbonyl cyanide *p*-trifluoromethoxyphenylhydrazone), and 1 µmol/l Rotenone. Open circles: pcDNA3.1. Open squares: Hdac7. Closed circles: pcDNA3.1 + MC1568. Closed squares: Hdac7 + MC1568. **d, e** Glucose-stimulated mitochondrial OCR (mOCR) was significantly decreased in Hdac7-overexpressing cells compared with control cells, when measured both in absolute values (**d**) and as fold change [ratio between glucose-stimulated and basal respiration (**e**)]. Adding MC1568 normalized mOCR in Hdac7-overexpressing cells (*n* = 6). **f** Cellular ATP levels at 16.7 mM glucose were reduced in Hdac7*-*overexpressing clonal β-cells compared with control cells and this was partly reversed by treatment with MC1568 (*n* = 6). **g** Overexpression of Hdac7 for 72 h in clonal β-cells resulted in reduced cell numbers when compared with control cells. This was normalized after treatment with 1 µmol/l MC1568 (*n* = 6). **h** Hdac7-overexpressing clonal β-cells showed increased apoptosis compared with control cells. MC1568 treatment resulted in a complete normalization of apoptosis (*n* = 6)

In an independent set of experiments, we could confirm that MC1568 treatment rescues the insulin secretion defect induced by Hdac7 overexpression in clonal β-cells (Fig. 1a, b) [10]. MC1568 also rescued the Hdac7-associated defects in mitochondrial respiration (Fig. 1c, d, e). Glucose-stimulated respiration (Fig. 1d) and the response to glucose elevation (the ratio between glucose-stimulated and basal mitochondrial respiration, Fig. 1e) were both the same in control and Hdac7-transfected cells treated with MC1568. These changes were mirrored by an increased ATP content in Hdac7 overexpressing β-cells stimulated with 16.7 mmol/l glucose after MC1568 treatment (Fig. 1f). In addition, MC1568 treatment reversed the negative effects of Hdac7 overexpression on β-cell number and apoptosis (Fig. 1g, h).

### MC1568 improves insulin secretion in islets from humans with T2D

**Fig. 2:**
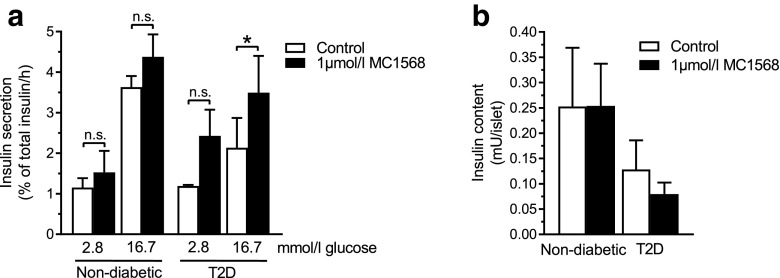
MC1568 improves glucose-stimulated insulin secretion in islets from donors with T2D. **a** Treatment with MC1568 had no effect on insulin secretion in islets from non-diabetic donors (*n* = 3), while it resulted in increased GSIS in islets from donors with T2D (*n* = 3). *p < 0.05. **b** MC1568 had no effect on insulin content in islets from either non-diabetic donors or donors with T2D. Data are presented as mean ± SEM. *p < 0.05, *n.s*. not significant

We next tested if MC1568 treatment could increase in vitro glucose-stimulated insulin secretion (GSIS) in human islets from non-diabetic donors and donors with T2D (Table 1). Static islet incubations showed that MC1568 had no significant effect on either basal or glucose-stimulated insulin secretion in islets from non-diabetic donors (Fig. 2a). However, islets from donors with T2D showed significantly increased GSIS after treatment with MC1568 (*p* < 0.05), while the effect on basal secretion was not statistically significant (Fig. 2a). In addition, MC1568 increased secretion at high glucose for all three donors with T2D individually (*p* < 0.05), while it did not for any of the non-diabetic donors (data not shown). Insulin content was not affected by the inhibitor (Fig. 2b).

**Table 1:** Characteristics of human pancreatic islet donors

Characteristic	Non-diabetics	T2D
Sex (W/M)	1W/2M	1W/2M
Age (years)	61.0 ± 23.8	58.7 ± 4.0
BMI (kg/m^2^)	25.8 ± 2.2	27.8 ± 2.4
HbA1c (%)	5.8 ± 0.5	6.2 ± 0.4
HbA1c (mmol/mol)	39.3 ± 5.0	44.0 ± 4.4

Mean ± SD

## Discussion

Our study demonstrates that a selective class II HDAC inhibitor, MC1568, improves insulin secretion in human islets from donors with T2D. It also rescued mitochondrial dysfunction and apoptosis in clonal β-cells overexpressing Hdac7. These findings support specific HDAC7 inhibitors as a potential therapeutic option for T2D.

We recently found increased *HDAC7* expression in islets from donors with T2D and *HDAC7* expression correlated negatively with GSIS. These data suggested that HDAC7 may impair insulin secretion [10]. Indeed, Hdac7 overexpression impaired insulin secretion in both rat islets and clonal β-cells, and resulted in increased expression of *Tcf7l2* and decreased expression of gene sets regulating DNA replication and repair as well as nucleotide metabolism in β-cells. Moreover, the impaired insulin secretion mediated by Hdac7 overexpression was restored by MC1568 treatment [10]. To translate these findings to humans, we tested if MC1568 could improve insulin secretion in islets from human donors with T2D. Indeed, MC1568 increased GSIS in T2D islets, but had no effect on islets from non-diabetic donors. We very rarely receive islets from donors with T2D, and these unique data have been collected over a long time. However, islets from a larger number of donors should be investigated in the future to further strengthen this notion. In addition, due to the limited access to islets from donors with T2D, we could not investigate the cellular effects contributing to this improvement in insulin secretion by MC1568. Instead, to dissect the cellular mechanisms by which Hdac7 inhibition rescues the insulin secretion impairment, we treated Hdac7 overexpressing β-cells with MC1568. We found that HDAC inhibition improved the mitochondrial oxygen consumption rate and cellular ATP levels at high glucose levels in Hdac7-overexpressing cells, which support an improved mitochondrial function. These data are in line with a previous study reporting that HDAC inhibition improves oxidative metabolism and mitochondrial function in muscle cells and adipocytes [11]. In addition, a previous study found changes in histone acetylation when oocytes were exposed to MC1568 for several relatively short time points, supporting that short exposures to MC1568 can alter the acetylation of histones [12].

Hdac7 overexpression, i.e., mimicking the T2D situation, in clonal β-cells also resulted in increased cell death. Studies indicate that T2D is associated with β-cell loss due to various mechanisms, at least in advanced stages of the disease [13], and increased HDAC7 may be a contributing factor in this. Our data suggest that MC1568 can prevent β-cell death induced by Hdac7 overexpression and, thus, rescue β-cell numbers. Interestingly, and in agreement with our finding, treatment with MC1568 in pancreatic explants from mice increased the pool of β- and δ-cells [8].

In summary, HDAC7 inhibition protects β-cells from mitochondrial dysfunction and apoptosis, and increases glucose-stimulated insulin secretion in islets from human T2D donors. Our study supports specific HDAC7 inhibitors as novel options in the treatment of T2D.

## Abbreviations

FCCP: Carbonyl cyanide *p*-trifluoromethoxyphenylhydrazone
BR: Basal respiration
GSR: Glucose-stimulated respiration
HDAC: Histone deacetylase
mOCR: Mitochondrial oxygen consumption rate
Oligo: Oligomycin
OCR: Oxygen consumption rate
SAB: Secretion assay buffer
T2D: Type 2 diabetes

## References

[1] Ling C, Groop L (2009). Epigenetics: a molecular link between environmental factors and type 2 diabetes. Diabetes.

[2] Volkov P, Bacos K, Ofori JK (2017). Whole-genome bisulfite sequencing of human pancreatic islets reveals novel differentially methylated regions in type 2 diabetes pathogenesis. Diabetes.

[3] Chen WB, Gao L, Wang J (2016). Conditional ablation of HDAC3 in islet beta cells results in glucose intolerance and enhanced susceptibility to STZ-induced diabetes. Oncotarget.

[4] Chou DH, Holson EB, Wagner FF (2012). Inhibition of histone deacetylase 3 protects beta cells from cytokine-induced apoptosis. Chem Biol.

[5] Lundh M, Galbo T, Poulsen SS, Mandrup-Poulsen T (2015). Histone deacetylase 3 inhibition improves glycaemia and insulin secretion in obese diabetic rats. Diabetes Obes Metab.

[6] Remsberg JR, Ediger BN, Ho WY (2017). Deletion of histone deacetylase 3 in adult beta cells improves glucose tolerance via increased insulin secretion. Mol Metab.

[7] Wagner FF, Lundh M, Kaya T (2016). An isochemogenic set of inhibitors to define the therapeutic potential of histone deacetylases in beta-cell protection. ACS Chem Biol.

[8] Lenoir O, Flosseau K, Ma FX (2011). Specific control of pancreatic endocrine beta- and delta-cell mass by class IIa histone deacetylases HDAC4, HDAC5, and HDAC9. Diabetes.

[9] Khan S, Jena G (2016). Valproic acid improves glucose homeostasis by increasing beta-cell proliferation, function, and reducing its apoptosis through HDAC inhibition in juvenile diabetic rat. J Biochem Mol Toxicol.

[10] Daneshpajooh M, Bacos K, Bysani M (2017). HDAC7 is overexpressed in human diabetic islets and impairs insulin secretion in rat islets and clonal beta cells. Diabetologia.

[11] Galmozzi A, Mitro N, Ferrari A (2013). Inhibition of class I histone deacetylases unveils a mitochondrial signature and enhances oxidative metabolism in skeletal muscle and adipose tissue. Diabetes.

[12] Wang H, Cui W, Meng C (2018). MC1568 enhances histone acetylation during oocyte meiosis and improves development of somatic cell nuclear transfer embryos in pig. Cell Reprogram.

[13] Halban PA, Polonsky KS, Bowden DW (2014). β-cell failure in type 2 diabetes: postulated mechanisms and prospects for prevention and treatment. Diabetes Care.

